# Frequency and clinicopathological features of metastasis to liver, lung, bone, and brain from gastric cancer: A SEER‐based study

**DOI:** 10.1002/cam4.1661

**Published:** 2018-07-09

**Authors:** Miao‐Zhen Qiu, Si‐Mei Shi, Zhan‐Hong Chen, Hong‐En Yu, Hui Sheng, Ying Jin, De‐Shen Wang, Feng‐Hua Wang, Yu‐Hong Li, Dan Xie, Zhi‐Wei Zhou, Da‐Jun Yang, Rui‐Hua Xu

**Affiliations:** ^1^ Department of Medical Oncology State Key Laboratory of Oncology in South China Collaborative Innovation Center for Cancer Medicine Sun Yat‐Sen University Cancer Center Guangzhou China; ^2^ Department of Nursing State Key Laboratory of Oncology in South China Collaborative Innovation Center for Cancer Medicine Sun Yat‐Sen University Cancer Center Guangzhou China; ^3^ Department of Experimental Research State Key Laboratory of Oncology in South China Collaborative Innovation Center for Cancer Medicine Sun Yat‐Sen University Cancer Center Guangzhou China; ^4^ Department of Gastric Surgery State Key Laboratory of Oncology in South China Collaborative Innovation Center for Cancer Medicine Sun Yat‐Sen University Cancer Center Guangzhou China

**Keywords:** gastric cancer, metastases, SEER

## Abstract

The hematogenous metastatic pattern of gastric cancer (GC) was not fully explored. Here we analyzed the frequency and clinicopathological features of metastasis to liver, lung, bone, and brain from GC patients. Data queried for this analysis included GC patients from the Surveillance, Epidemiology, and End Results Program database from 2010 to 2014. All of statistical analyses were performed using the Intercooled Stata 13.0 (Stata Corporation, College Station, TX). All statistical tests were two‐sided. Totally, there were 19 022 eligible patients for analysis. At the time of diagnosis, there were 7792 patients at stage IV, including 3218 (41.30%) patients with liver metastasis, 1126 (14.45%) with lung metastasis, 966 (12.40%) with bone metastasis and 151 (1.94%) with brain metastasis. GC patients with lung or liver metastasis have a higher risk of bone and brain metastasis than those without lung nor liver metastasis. Intestinal subtype had significantly higher rate of liver and lung metastasis, while diffuse type was more likely to have bone metastasis. Proximal stomach had significantly higher risk to develop metastasis than distal stomach. African‐Americans had the highest risk of liver metastasis and Caucasian had the highest prone to develop lung and brain metastasis. The median survival for patients with liver, lung, bone, and brain metastasis was 4 months, 3 months, 4 months and 3 months, respectively. It is important to evaluate the status of bone and brain metastasis in GC patients with lung or liver metastasis. Knowledge of metastatic patterns is helpful for clinicians to design personalized pretreatment imaging evaluation for GC patients.

## 1 INTRODUCTION

Gastric cancer (GC) was a leading cause of cancer‐related deaths in the early 20th century but both incidence and mortality rates have steadily declined over the last century in the United States.^1^, ^2^ GC is still the second most common cause of cancer‐related death in developing countries.^3^, ^4^, ^5^, ^6^, ^7^, ^8^, ^9^, ^10^ The estimated new GC patients in United States in 2017 are 28 000 and estimated deaths are 10 960.^11^ Almost one‐third of GC were diagnosed at stage IV.^12^

From 2010 on, the Surveillance, Epidemiology and End Results (SEER) data started to announce metastatic pattern including liver, lung, bone, and brain. The major feature of recurrence in GC patients is intraabdominal spread.^13^ Liver is the most common site of hematogenous metastasis of GC.^14^, ^15^ Lung metastasis is reported to be around 0.5%‐0.96% in GC patients.^16^, ^17^, ^18^ Bone metastasis by GC is rare, occurring in only 0.9%‐3.8% of gastric cancer patients.^19^, ^20^, ^21^, ^22^ Brain metastasis from GC is relatively rare and the incidence rate is about 0.16%‐0.69%.^23^, ^24^ Most reports on bone and brain metastasis from GC are case reports. Due to the limitation of sample size, the incidence rate of metastasis to above sites may not be estimated sufficiently.

In this study, we used data from the SEER cancer‐registry program of individuals diagnosed with GC from 2010 to 2014 to analyze the metastatic pattern. Knowledge of metastatic distribution may help physicians to design imagine examination, especially in making determinations regarding curative‐intent interventions.

## 2 METHODS

### 2.1 Database

The SEER database is the largest publicly available cancer dataset. It is a population‐based cancer registry across several disparate geographic regions. The SEER research data include cancer incidence and prevalence as well as demographic information tabulated by age, sex, race/ethnicity, year of diagnosis, marital status, insurance, Tumor‐Node‐Metastasis (TNM) stage and geographic region. The exact dataset we used for this analysis was SEER Program (http://www.seer.cancer.gov) Research Data (1973‐2014), National Cancer Institute, DCCPS, Surveillance Research Program, Surveillance Systems Branch, based on the November 2016 submission. The SEER database started to release metastatic information related to liver, lung, bone and brain in 2010. On 7 December 2017, the SEER database just released US Mortality databases including 2015 deaths. Therefore it is possible to analyze the 5 year cause specific survival for patients from 2010 on using the SEER database.

### 2.2 Outcome variables

Variable definitions information on age at diagnosis, sex, year of diagnosis, race/ethnicity, marital status, primary site, tumor grade and differentiation, histology, lymph node involvement, AJCC 7th TNM stage, insurance status and overall survival were coded and available in SEER database.

The primary site was defined by the following International Classification of Diseases for Oncology (ICD‐O‐2) codes: cardia, (C16.0), fundus (C16.1), body (C16.2), antrum (C16.3), pylorus (C16.4), lesser curvature (C16.5), greater curvature (C16.6), overlapping lesion (C16.7) and stomach, NOS (C16.9).

Grade and differentiated was defined by the following ICD‐O‐2 codes; well differentiated (Code 1), moderate differentiated (Code 2), poorly differentiated (Code 3) and undifferentiated (Code 4).

Histological types were defined by the following ICD‐O‐3 codes: 8140 to 8147, 8210 to 8211, 8220 to 8221, and 8260 to 8263 for adenocarcinoma, 8480 and 8481 for mucinous adenocarcinoma, and 8490 for Signet ring cell carcinoma.

For the Race/Ethnicity, we reclassified patients into five groups: “Caucasian,” “African‐American,” “Asian,” “Others,” and “Unknown.”

For the insurance status, individuals in the “Any Medicaid,” “Insured,” and “Insured/No specifics” groups were clustered together as “Insured group.” Patients were therefore divided into “insured group” and “uninsured group”.

Patients were classified as married and unmarried. Since the group of “Unmarried or domestic partner” is misleading and we removed this group of patients from analysis. Unmarried patients included single, separated/divorced, and widowed.

### 2.3 Patient population

The study population was based on the SEER cancer registry. We restricted eligibility to patients with gastric adenocarcinoma (including mucinous adenocarcinoma and signet ring cell carcinoma) from 2010 to 2014. We excluded cases without records of follow‐up (survival time code of 0 months) and TNM stage.

### 2.4 Statistical methods

The patients’ demographic and tumor characteristics were summarized with descriptive statistics. Comparisons of categorical variables were performed using the Chi square test, and continuous variables were compared using Student's *t* test. The primary endpoint of this study was 5‐year cause specific survival (CSS), which was calculated from the date of diagnosis to the date of cancer specific death. Deaths attributed to gastric cancer were treated as events and deaths from other causes were treated as censored observations. Survival function estimation and comparison among different variables were performed using Kaplan‐Meier estimates and the log‐rank test. The independence of the prognostic factors was adjusted for other known factors including age at diagnosis and tumor stage. All of statistical analyses were performed using the Intercooled Stata 13.0 (Stata Corporation, College Station, TX). Statistical significance was set at two‐sided *P *<* *0.05.

### 2.5 Informed consent

This study was deemed exempt from institutional review board approval by Sun Yat‐sen University Cancer Center and informed consent was waived.

## 3 RESULT

### 3.1 Patient characteristics

The study group consisted of 19 022 patients, including 12 208 men (64.18%) and 6814 women (35.82%). The median age of the whole group was 66 years old. The distribution of AJCC 7th TNM stage from I to IV were 21.63%, 14.92%, 22.48%, and 40.96%, respectively.

### 3.2 Metastasis pattern

At the time of diagnosis, there were 3218 (16.92%) patients with liver metastasis, 1126 (5.92%) patients with lung metastasis, 966 (5.08%) patients with bone metastasis and 151 (0.79%) patients with brain metastasis. Patients who had metastasis to either one of the four sites accounted for 60.73% (4732/7792) of stage IV diseases. Clinical features of GC patients were presented in Table 1.

**Table 1:** Clinical features and metastasis sites for gastric cancer

Features	Liver (%)		*P* a	Lung (%)		*P* b	Bone (%)		*P* c	Brain (%)		*P* d
	0	1		0	1		0	1		0	1	
Sex												
Women	5753 (86.24)	918 (13.76)	<0.001	6276 (94.58)	360 (5.42)	0.006	6322 (95.18)	320 (4.82)	0.083	6590 (99.43)	38 (0.57)	0.007
Men	9703 (80.84)	2300 (19.16)		11148 (93.57)	766 (6.43)		11302 (94.59)	646 (5.41)		11816 (99.05)	113 (0.95)	
Age at diagnosis	65.74 ± 14.15	65.66 ± 13.20	0.785	65.82 ± 13.95	64.21 ± 14.52	0.0002	65.99 ± 13.93	60.68 ± 13.99	<0.001	65.75 ± 13.98	61.34 ± 13.64	0.0001
Married status												
Married	8786 (82.61)	1850 (17.39)	0.840	9925 (94.04)	629 (5.96)	0.142	10021 (94.71)	560 (5.29)	0.874	10482 (99.19)	86 (0.81)	0.798
Unmarried	5880 (82.72)	1228 (17.28)		6614 (93.50)	460 (6.50)		6712 (94.76)	371 (5.24)		7003 (99.15)	60 (0.85)	
Ethnicity												
Caucasian	10743 (82.21)	2325 (17.79)	<0.001	12128 (93.56)	835 (6.44)	<0.001	12300 (94.53)	712 (5.47)	0.151	12868 (99.02)	127 (0.98)	0.002
African‐American	1909 (80.18)	472 (19.82)		2234 (93.91)	145 (6.09)		2265 (95.45)	108 (4.55)		2356 (99.58)	10 (0.42)	
Asian	2413 (87.68)	339 (12.32)		2623 (95.76)	116 (4.24)		2614 (95.47)	124 (4.53)		2717 (99.49)	14 (0.51)	
Primary tumor sites												
Cardia	5003 (78.77)	1348 (21.23)	<0.001	5806 (92.13)	496 (7.87)	<0.001	5956 (94.20)	367 (5.80)	<0.001	6235 (98.75)	79 (1.25)	<0.001
Fundus	520 (76.36)	161 (23.64)		616 (91.26)	59(8.74)		652 (96.02)	27(3.98)		667 (98.23)	12 (1.77)	
Body	1440 (84.51)	264 (15.49)		1621 (95.69)	73 (4.31)		1603 (94.46)	94 (5.54)		1685 (99.59)	7 (0.41)	
Antrum	3112 (87.54)	443 (12.46)		3427 (96.81)	113 (3.19)		3436 (97.17)	100 (2.83)		3526 (99.77)	8 (0.23)	
Pylorus	425 (87.45)	61 (12.55)		465 (96.67)	16(3.33)		469 (97.10)	14(2.90)		480 (99.59)	2 (0.41)	
Lesser curvature	1255 (87.95)	172 (12.05)		1367 (96.34)	52 (3.66)		1373 (96.55)	49 (3.45)		1415 (99.58)	6 (0.42)	
Greater curvature	559 (85.21)	97 (14.79)		624 (95.41)	30(4.59)		623 (95.70)	28(4.30)		650 (99.39)	4 (0.61)	
Overlapping	1248 (85.25)	216 (14.75)		1380 (95.11)	71 (4.89)		1387 (94.74)	77 (5.26)		1457 (99.59)	6 (0.41)	
Histology												
Adenocarcinoma	11459 (79.74)	2912 (20.26)	<0.001	13322 (93.5)	926 (6.5)	<0.001	13624 (95.47)	646 (4.53)	<0.001	14134 (99.18)	117 (0.82)	<0.001
Mucinous	327 (86.28)	52 (13.72)		350 (93.09)	26(6.91)		362 (94.52)	21(5.48)		376 (99.47)	2 (0.53)	
Signet ring cell carcinoma	3670 (93.53)	254 (6.47)		3752 (95.57)	174 (4.43)		3638 (92.41)	299 (7.59)		3896 (99.19)	32 (0.81)	
Grade												
Well	699 (91.25)	67 (8.75)	<0.001	735 (96.2)	29 (3.8)	<0.001	757 (98.95)	8 (1.05)	<0.001	766 (100)	0 (0)	<0.001
Moderately	3487 (79.67)	890 (20.33)		4075 (93.94)	263 (6.06)		4225 (97.28)	118 (2.72)		4300 (99.01)	43 (0.99)	
Poorly	8801 (84.78)	1580 (15.22)		9753 (94.57)	560 (5.43)		9752 (94.32)	587 (5.68)		10258 (99.38)	64 (0.62)	
Undifferentiated	243 (90.33)	26 (9.67)		257 (95.9)	11 (4.1)		262 (97.4)	7 (2.60)		266 (99.63)	1 (0.37)	
Lauren												
Diffuse	4745 (93.46)	332 (6.54)	<0.001	4857 (95.8)	213 (4.20)	<0.001	4742 (93.25)	343 (6.75)	<0.001	5039 (99.33)	34 (0.67)	0.220
Intestinal	10151 (78.30)	2814 (21.70)		11973 (93.15)	880 (6.85)		12272 (95.35)	599 (4.65)		12741 (99.11)	114 (0.89)	
Insurance												
Insured	14472 (82.92)	2980 (17.08)	0.017	16294 (94.00)	1040 (6.00)	0.052	16490 (94.91)	884 (5.09)	0.001	17206 (99.22)	136 (0.78)	0.219
Uninsured	669 (79.17)	176 (20.83)		777 (92.06)	67(7.94)		776 (92.05)	67(7.95)		830 (98.69)	11 (1.31)	
Surgery												
Yes	8434 (97.28)	236 (2.72)	<0.001	8611 (99.39)	53 (0.61)	<0.001	8625 (99.65)	30 (0.35)	<0.001	8648 (99.86)	12 (0.14)	<0.001
No	6827 (69.70)	2968 (30.30)		8608 (88.93)	1071 (11.07)		8796 (90.42)	932 (9.58)		9552 (98.57)	139 (1.43)	

NOS, not otherwise specified.

a The comparison between patients with and without liver metastasis.

b The comparison between patients with and without lung metastasis.

c The comparison between patients with and without bone metastasis.

d The comparison between patients with and without brain metastasis.

### 3.3 Liver metastasis

Male patients had higher percentage of liver metastasis than female. For ethnicity, African‐American patients had highest percentage (19.82%) of liver metastasis and Asian patients had lowest (12.32%). Primary tumor at the cardia and fundus had higher percentage of liver metastasis, while antrum, pylorus and lesser curvature had lower liver metastatic rate. Signet ring cell carcinoma had lowest rate of liver metastasis compared with adenocarcinoma and mucinous adenocarcinoma. For the tumor grade, moderately differentiated tumors had highest percentage (20.33%) of liver metastasis and well differentiated tumors had lowest (8.75%). In the Lauren classification, intestinal subtype had significantly higher rate of liver metastasis than diffuse type, 21.70% vs 6.54%, *P *<* *0.001. The metastatic rate in uninsured patients was significantly higher than insured patients, *P *=* *0.017. Age was not significantly different between patients with and without liver metastasis.

### 3.4 Lung metastasis

Features for patients with lung metastasis were similar to those with liver metastasis, including male predominant and more intestinal subtype. Patients with lung metastasis were significantly younger than those without. Caucasian patients had higher percentage of lung metastasis than African‐American and Asian patients. Mucinous adenocarcinoma patients had higher lung metastatic rate than adenocarcinoma and signet ring cell carcinoma.

### 3.5 Bone metastasis

There was no significant difference of bone metastasis between male and female. The median age of patients with bone metastasis was 6 years younger than those without. The bone metastatic rate was not significantly different among races. Cardia and body had higher percentages of bone metastasis, while antrum and pyrolus had lowest. Patients with signet ring cell carcinoma had higher percentage of bone metastasis than mucinous and adenocarcinoma. Poorly differentiated tumors had highest bone metastatic rate and well differentiated tumors had the lowest. For Lauren classification, diffuse type had higher bone metastatic rate than intestinal type.

### 3.6 Brain metastasis

The median age of patients with bone metastasis was 5 years younger than those without. Caucasian patients had higher percentage of brain metastasis than African‐American and Asian patients. Mucinous patients had lower percentage of brain metastasis than adenocarcinoma and signet ring cell carcinoma. Moderately differentiated tumors had higher brain metastatic rate than well, poorly, and undifferentiated tumors. There was no significant difference of brain metastasis between diffuse and intestinal type as well as insured and uninsured type.

### 3.7 Combination of metastasis patterns

Many patients developed more than one site of metastatic diseases. Table 2 summarized all the possible combinations of these four sites of metastasis. The most common two‐site metastasis combination was liver and lung (2.25%). Only 11 (0.06%) patients had all four sites metastasis.

**Table 2:** Frequencies of combination metastasis and 5‐y CSS

	Number (%)	5‐y CSS (95% CI)	Median OS (mo)
No metastasis	11 230 (59.04)	44.34% (42.47%‐46.19%)	37
One site			
Only liver	2247 (11.81)	5.19% (3.79%‐6.89%)	5
Only lung	396 (2.08)	3.26% (0.09%‐8.11%)	5
Only bone	487 (2.56)	0	4
Only brain	52 (0.27)	0	3
Two sites			
Lung and liver	428 (2.25)	2.04% (0.68%‐4.81%)	3
Lung and bone	92 (0.48)	4.71% (1.09%‐12.66%)	4
Lung and brain	12 (0.06)	9.09% (0.54%‐33.29%)	3
Liver and bone	172 (0.90)	1.49% (0.17%‐6.24%)	4
Liver and brain	18 (0.09)	0	1
Bone and brain	15 (0.08)	4.44% (0.08%‐25.96%)	2
Three sites			
Lung and liver and bone	102 (0.54)	5.08% (1.07%‐14.13%)	3
Lung and liver and brain	17 (0.09)	0	2
Liver and bone and brain	7 (0.04)	0	3
Lung and bone and brain	6 (0.03)	16.67% (0.77%‐51.68%)	1
Four sites			
Liver and lung and bone and brain	11 (0.06)	9.09% (0.54%‐33.29%)	2
Metastasis to other sites	3060 (16.09)	2.60% (1.14%‐5.10%)	7
Metastasis to unknown combination	670 (3.52)	2.77% (1.36%‐5.01%)	4

CSS, Cause Specific Survival; CI, confidence interval; OS, Overall survival.

**Figure 1:**
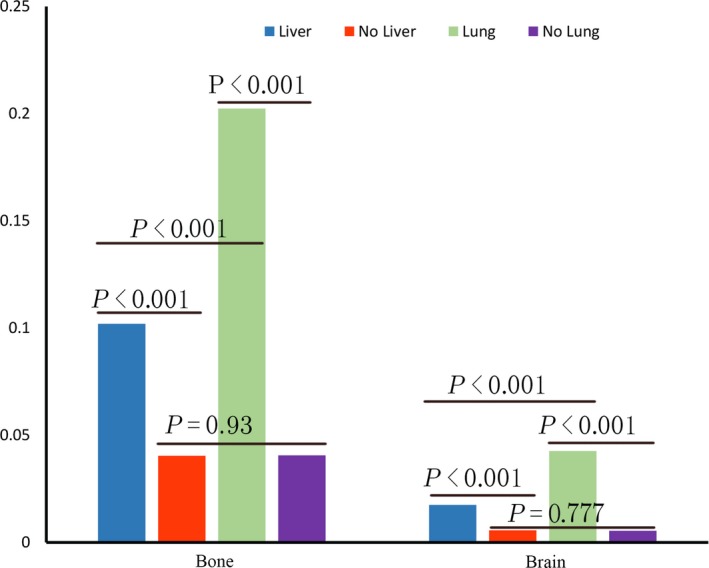
Comparisons of Metastatic Rate to Bone and Brain With and Without Liver or Lung Metastasis

Furthermore, we compared the risk of bone and brain metastasis between patients with and without lung or liver metastasis. We found that patients with lung metastasis had a higher risk of bone (20.24% vs 4.06%, *P *<* *0.001) or brain metastasis (4.27% vs 0.54%, *P *<* *0.001) than patients without (Figure 1). Though a similar phenomenon was noted for liver metastasis, with higher risk of bone (10.20% vs 4.04%, *P *<* *0.001) and brain metastasis (1.75% vs 0.57%, *P *<* *0.001) for liver metastasis patients than those without, GC patients with lung metastasis had a higher incidence rate of bone or brain metastasis than patients with liver metastasis.

### 3.8 Survival

**Figure 2:**
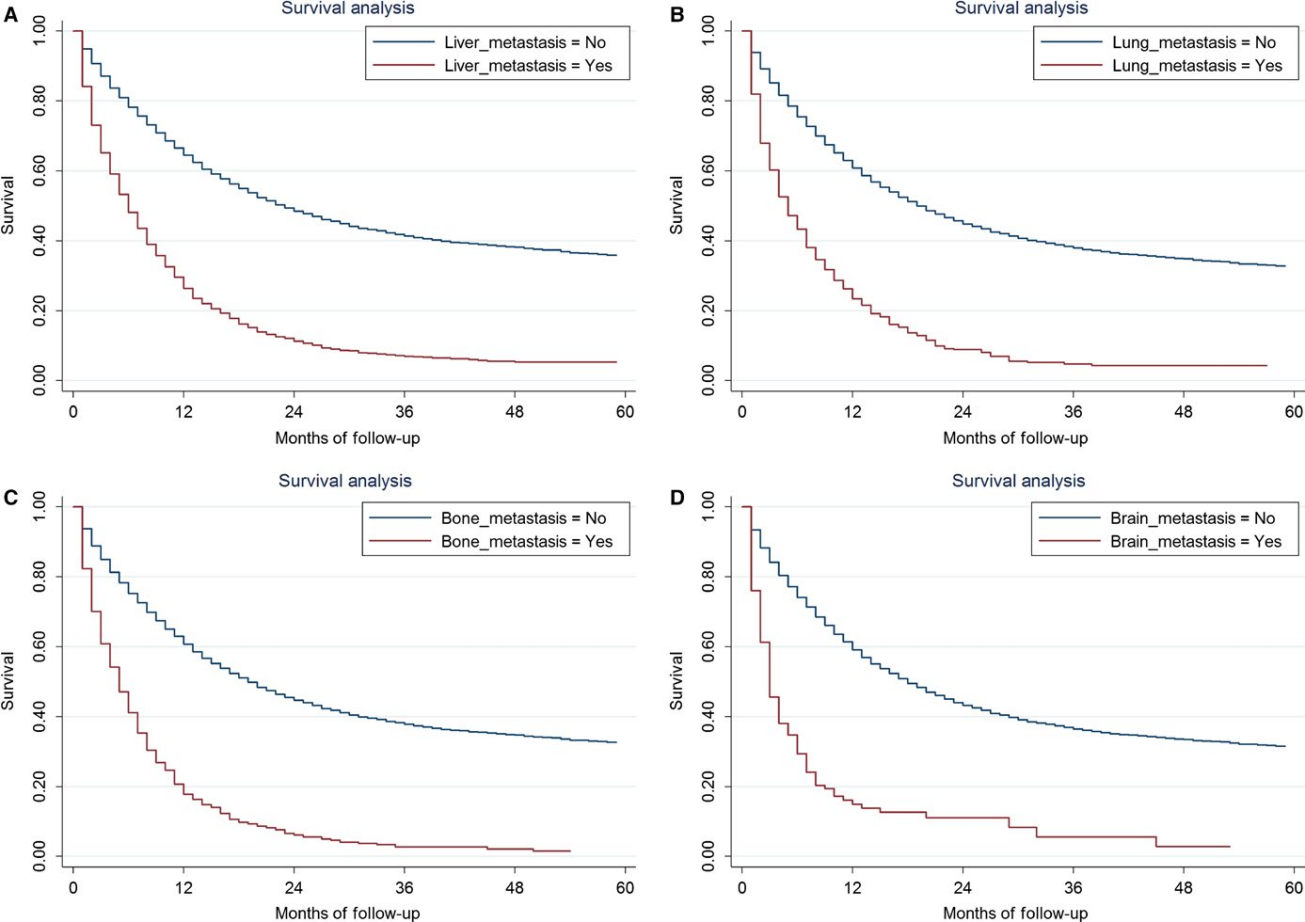
Cause Specific Survival between Patients With and Without Liver Metastasis, *P *<* *0.001 (A), with and without lung metastasis, *P *<* *0.001 (B), with and without bone metastasis, *P *<* *0.001 (C), with and without brain metastasis, *P *<* *0.001 (D)

In this study, 10 029 deaths (10.56%) were observed. The 5‐year CSS was 27.77% for the whole cohort, with a median OS of 14 months. The 5‐year CSS was 4.28% vs 33.24% for patients with and without liver metastasis (*P *<* *0.001), 3.35% vs 30.01% for patients with and without lung metastasis (*P *<* *0.001), 1.27%% vs 29.86% for patients with and without bone metastasis (*P *<* *0.001) and 2.32% vs 28.68% for patients with and without brain metastasis (*P *<* *0.001) (Figure 2). The median OS for patients with liver, lung, bone, and brain metastasis was 4 months, 3 months, 4 months, and 3 months, respectively. The median OS and 5‐year CSS for patients with different combination of metastasis was showed in Table 2.

### 3.9 Prognostic factors for GC patients with liver metastasis

Since liver metastasis is the most common site of hematogenous metastasis. We analyzed the prognostic factors for liver metastasis from GC patients. Variables showing a trend for association with survival (*P *<* *0.05) in univariated analysis were selected in the cox proportional hazards model. Sex, age, married status, location, Lauren classification, histology, grade, insurance, and surgery were selected in the multivariate analysis. Age, marital status, histologic subtypes, insurance, and surgery were all independent prognostic factors in the multivariable analysis (Table 3).

**Table 3:** Survival analysis for GC patients with liver metastasis

	Univariate analysis			Multivariate analysis		
	Median OS (mo)	5‐y CSS (95% CI)	*P* value	Hazard ratio	95% CI	*P* value
Sex						
Male	5	4.09% (2.89%‐5.60%)		Reference		
Female	4	4.84% (2.97%‐7.36%)	0.044	1.022	0.93‐1.13	0.662
Age						
<66	6	4.50% (2.92%‐6.57%)		Reference		
>65	3	4.00% (2.73%‐5.63%)	<0.001	1.276	1.17‐1.40	<0.001
Married status						
Married	5	4.28% (3.01%‐5.88%)		Reference		
Unmarried	3	3.72% (2.24%‐5.78%)	<0.001	1.239	1.13‐1.36	<0.001
Location						
Cardia	6	2.98% (1.66%‐4.92%)		Reference		
Fundus	3	7.02% (2.77%‐14.00%)		0.932	0.75‐1.15	0.515
Body	4	5.50% (2.43%‐10.41%)		1.042	0.87‐1.24	0.648
Antrum	4	8.18% (5.08%‐12.19%)		1.059	0.92‐1.22	0.428
Pylorus	4	7.26% (1.74%‐18.33%)		0.958	0.68‐1.35	0.808
Lesser curvature	6	8.19% (3.18%‐18.06%)		0.870	0.71‐1.07	0.193
Greater curvature	2	1.8% (0.16%‐8.25%)		1.390	1.06‐1.83	0.019
Overlapping	2	0 (0)		1.481	1.23‐1.78	<0.001
NOS	3	2.65% (0.9%‐6.09%)	<0.001	1.255	1.10‐1.44	0.001
Race/Ethnicity						
Caucasian	5	4.13% (2.94%‐5.62%)		—		
African‐American	4	4.32% (1.88%‐8.35%)		—		
Asian	5	4.53% (2.08%‐8.42%)		—		
Others	4	4.20% (0.44%‐15.65%)	0.9216	—		
Lauren						
Diffuse	4	2.30% (0.58%‐6.32%)		Reference		
Intestinal	5	4.51% (3.37%‐5.89%)	0.0144	0.87	0.66‐1.16	0.351
Histology subgroup						
Adenocarcinoma	5	4.62% (3.49%‐5.97%)		Reference		
Signet Ring cell	4	1.15% (0.11%‐5.30%)		1.20	1.02‐1.41	0.029
Mucinous adenocarcinoma	3	2.40% (0.19%‐10.79%)	0.0012	1.57	1.14‐2.18	0.006
Grade						
Well differentiated	4	3.59% (0.32%‐14.52%)		Reference		
Moderately differentiated	6	6.43% (4.18%‐9.31%)		0.81	0.59‐1.13	0.213
Poorly differentiated	4	3.26% (2.04%‐4.94%)		1.05	0.76‐1.45	0.777
Undifferentiated	3	0 (0)	<0.001	0.83	0.46‐1.49	0.527
Insurance						
Insured	5	4.45% (3.36%‐5.74%)		Reference		
Uninsured	3	2.45% (0.25%‐10.10%)	0.0262	1.31	1.07‐1.61	0.01
Surgery						
Yes	9	17.74% (12.18%‐24.16%)		Reference		
No	4	2.95% (1.97%‐4.23%)	<0.001	1.82	1.52‐2.18	<0.001

OS, Overall survival; CSS, Cause Specific Survival; CI, Confidence Intervals; NOS, Not otherwise specified.

## 4 DISCUSSION

In our study, the metastatic rate to liver, lung, bone and brain from GC patients at the time of diagnosis was 16.92%, 5.92%, 5.08%, and 0.79%, respectively. The metastatic rate to above four sites were much higher than previous literature reports, which were 13.5% for liver metastasis,^15^ 0.5%‐0.96% for lung metastasis,^16^, ^17^, ^18^ 0.9%‐3.8% for bone metastasis^19^, ^20^, ^21^, ^22^ and 0.16%‐0.69% for brain metastasis.^23^, ^24^ The actual frequency of metastasis to bone and brain originating from GC was underestimated because usually bone and brain imaging was not performed as a routine evaluation and asymptomatic metastasis might be overlooked.^25^, ^26^ In our study, we found that the frequency of bone metastasis was similar to lung metastasis. More attention should be paid to evaluate status of metastasis to above sites using appropriate modalities. However it is inappropriate to recommend all the GC patients to screen metastasis to above sites, especially brain metastasis whose metastatic rate was less than 1%. It is feasible to find out the risk factors for metastasis. We found that patients with lung or liver metastasis have a higher risk of bone and brain metastasis than those without lung nor liver metastasis. Our previous study showed similar phenomenon in colorectal cancer.^27^ The internal mechanism of this distribution remains unknown. Primary lung cancer frequently metastasizes to bone as well as brain.^28^, ^29^, ^30^, ^31^ Some studies had suggested that there was cross‐talk among lung tumor cells, bone microenvironment, and immune system, which led to bone metastasis formation in primary non‐small cell lung cancer patients.^32^ This finding is helpful for us to design screen strategy. GC patients with lung or liver metastasis had higher risk of brain and bone metastasis. Therefore it is important to evaluate status of bone and brain metastasis in patients with lung or liver metastasis.

Our analysis found that intestinal subtype had significantly higher rate of liver and lung metastasis while diffuse type was more likely to have bone metastasis. Previous studies showed that liver metastases were more frequent in the intestinal type, while diffuse type had a greater propensity to metastasize to distant organs, including peritoneal dissemination.^9^, ^33^ Moderately differentiated tumors were more likely to have liver, lung, and brain metastasis, and poorly differentiated tumors had a prone to develop bone metastasis. The most common histopathological subtype for bone metastasis from GC was adenocarcinoma (79%) with poor differentiation (60.8%).^22^ Uninsured patients had higher percentages of liver and bone metastasis than insured patients. We did not found related reports in the literatures, while previous reports showed that insured GC patients were more likely to receive surgery and had better prognosis than uninsured.^34^ We therefore guessed that insured patients might receive more early intervention of GC and have a lower risk to develop metastatic diseases. Moreover, patients with proximal stomach cancer had significantly higher risk to develop metastasis than those with distal stomach cancer. For ethnicity, Asian patients had the lowest possibility to develop metastasis, while African‐Americans had the highest risk of liver metastasis and Caucasian had the highest prone to develop lung and brain metastasis. The relationship between tumor location, ethnicity and metastasis was not clearly described in the literatures. Our findings are helpful for clinicians to design personalized examinations for GC patients.

The outcome for GC patients with metastasis was poor, which were 4 months, 3 months, 4 months, and 3 months for metastasis to liver, lung, bone, and brain, respectively. The 5‐year survival for patients with liver metastasis was 11.4% in Japanese GC patients.^35^ The median survival times of 3‐4 months after detection of bone metastasis have been reported in some studies^36^, ^37^ and the median survival after treatment of brain metastasis was about 3 months.^23^ The univariated and multivariate analysis showed that younger patient, married, well and moderately differentiated tumor and surgery were related with better prognosis for GC patients with liver metastasis. Our previous studies showed that married GC patients were at lower risk of cancer specific mortality with the possibility that spouse might provide social supports and encourage the patients to seek medical treatments.^38^

To our knowledge, this is the first SEER‐based study focusing solely on the hematogenous metastatic pattern of GC patients. However, there are obvious limitations due to the retrospective nature of this study, as outlined below. First of all, it is important to note that the database only provide metastatic data to above 4 sites from 2010 on and the follow‐up time is not long enough. Moreover, we only have information on synchronous metastasis to liver, lung, bone, and brain, a relative minority compared to those patients who may develop metachronous metastasis. Furthermore, we don't have information of metastasis to other sites, especially the peritoneal metastasis. Overall, this is the first study to confirm the strong potential for bone and brain metastasis in patients who already have lung or liver metastasis. Based on our finding, we suggest that clinicians take the clinicopathological features into account when designing diagnostic and treatment algorithms.

## 5 CONCLUSIONS

It is important to evaluate status of bone and brain metastasis in GC patients with lung or liver metastasis. Knowledge of metastatic patterns is helpful for clinicians to design personalized pretreatment imaging evaluation for GC patients.

## CONFLICTS OF INTEREST

None declared.
